# The Tomato Feruloyl Transferase FHT Promoter Is an Accurate Identifier of Early Development and Stress-Induced Suberization

**DOI:** 10.3390/plants12091890

**Published:** 2023-05-05

**Authors:** Anurag Kashyap, Álvaro Jiménez-Jiménez, Mercè Figueras, Olga Serra, Marc Valls, Nuria S. Coll

**Affiliations:** 1 Centre for Research in Agricultural Genomics (CRAG), CSIC-IRTA-UAB-UB, Campus UAB, 08193 Bellaterra, Spain; 2 Laboratori del Suro, Biology Department, University of Girona, Campus Montilivi, 17003 Girona, Spain; 3 Department of Genetics, Universitat de Barcelona, 08028 Barcelona, Spain; 4 Consejo Superior de Investigaciones Científicas (CSIC), 08001 Barcelona, Spain

**Keywords:** suberin, ferulates, stress, development, tomato

## Abstract

As a wall polymer, suberin has a multifaceted role in plant development and stress responses. It is deposited between the plasma membrane and the primary cell wall in specialized tissues such as root exodermis, endodermis, phellem, and seed coats. It is formed de novo in response to stresses such as wounding, salt injury, drought, and pathogen attack and is a complex polyester mainly consisting of fatty acids, glycerol, and minor amounts of ferulic acid that are associated to a lignin-like polymer predominantly composed of ferulates. Metabolomic and transcriptomic studies have revealed that cell wall lignification precedes suberin deposition. The ferulic acid esterified to ω-hydroxy fatty acids, synthetized by the feruloyl transferase FHT (or ASFT), presumably plays a role in coupling both polymers, although the precise mechanism is not understood. Here, we use the promoter of tomato suberin feruloyl transferase (FHT/ASFT) fused to GUS (β-glucuronidase) to demonstrate that ferulate deposition agrees with the site of promoter FHT activation by using a combination of histochemical staining and UV microscopy. Hence, FHT promoter activation and alkali UV microscopy can be used to identify the precise localization of early suberizing cells rich in ferulic acid and can additionally be used as an efficient marker of early suberization events during plant development and stress responses. This line can be used in the future as a tool to identify emerging suberization sites via ferulate deposition in tomato plants, which may contribute to germplasm screening in varietal improvement programs.

## 1. Introduction

The wall polymer suberin has a multidimensional role in plant development and stress responses [1,2]. Structurally, it is a glycerol-based, fatty-acid-derived polyester comprised primarily of ω-hydroxy acids, α, ω-dicarboxylic acids, fatty alcohols, and small amounts of hydroxycinnamic acids (mainly ferulic acid) [3]. It is deposited between the plasma membrane and the cell wall in specialized tissues, such as root exodermis, endodermis, seed coat, and phellem (cork), in root and aboveground tissue in woody plants [4]. Moreover, suberin can be formed de novo in response to stress such as wounding, salt injury, drought, and pathogen attack [5]. In addition to providing strength to the cell wall, suberin averts water loss and pathogen access by sealing off the layer of suberized cells. It acts as a potent barricade against pathogens, and in addition to providing strength to the cell wall, it may also act as an antimicrobial fence [6]. Recently, it has been shown that microbiota inhabiting the roots can also influence suberin deposition at the endodermis [7]. Hence, suberin has great potential for developing crops with multi-stress tolerance [8]. The polymer is also being explored for carbon sequestration applications using plants as carbon storage to mitigate global warming. Suberized cell walls also accumulate a lignin-like polymer rich in ferulates in the primary cell wall, which has also led to them being called suberin polyphenolic domains [2]. However, the configuration of suberin and lignin polymers and the progression of their deposition are poorly understood [2]. Metabolomics and transcriptomics data suggest that lignification precedes suberization, and ferulic acid has been identified to initiate suberin deposition, possibly offering connections to lignin or other cell wall polymers [2,9]. Suberin biosynthesis occurring during developmental processes has been studied in the last two decades using molecular genetic methods, particularly in the endodermis and seed coat model of *Arabidopsis thaliana* and in the tuber phellem of potato [10]. Precursors of the lignin-like polymer originate from the phenylpropanoid pathway, as does the ferulic acid of suberin, while the aliphatic monomers of suberin are synthesized through the fatty acid biosynthetic pathway [6]. Several genes encoding enzymes involved in the modification of these fatty acid precursors yielding the suberin monomers have been described, such as fatty acyl-CoA reductase (FAR1, FAR4, FAR5); fatty acid cytochrome P450 oxidases (CYP86A1/HORST, CYP86B1, CYP94B1, CYP94B3); glycerol-3-phosphate acyltransferase 5 (GPAT5/GPAT7); β-ketoacyl-CoA synthases (KCS1, KCS2/Daisy, KCS6, and KCS20); and genes involved in the conveyance of suberin monomers to the site of suberization (ABCG1, ABCG2, ABCG6, ABCG11, and ABCG20) [11,12,13,14,15]. In addition, a few upstream controllers of the suberin biosynthetic pathway have been recognized in Arabidopsis, such as the MYB transcription factors MYB41, MYB107, and SUBERMAN (MYB39) [2,16,17,18]. In Arabidopsis, suberin feruloyl transferase (FHT, also known as ASFT/HHT/RWP) has been functionally characterized and has been reported to be involved in the incorporation of ferulic acid into the suberin polyester. It specifically controls the accumulation of the ferulate esters of suberin in root endodermis, seeds, and phellem, but it has no effect on the content of p-coumarate or sinapate [19,20,21]. Knocking out FHT/ASFT causes abolition of the suberin-ester-linked ferulate, leading to altered permeability and sensitivity of seeds and roots to salt stress [19,20], and its activation has been reported as one of the earliest steps in suberin deposition [21]. Recently, a set of four MYB transcription factors (MYB41, MYB53, MYB92, and MYB93), each of which is individually regulated by developmental and exogenous signals, was shown to be sufficient to promote endodermal suberin deposition [22]. Moreover, the transcription factor WRKY9 has been demonstrated to regulate cytochrome P450 genes CYP94B3 and CYP86B1, leading to increased root suberin and salt tolerance in Arabidopsis [23]. The role of NAC transcription factor ANAC046 in suberin biosynthesis in *Arabidopsis thaliana* roots has also been experimentally validated [24].

The tissues commissioning suberization undergo a series of genetic and metabolic reprogramming comprising a web of metabolic conduits to yield the precursors of the polymer and subsequently their polymerization into the matrix [25]. In tomato, suberin plays vital roles both as a constitutive barrier as well as an inducible fence against diverse environmental stresses [26]. However, the visualization of suberin in tomato tissues mostly relies on stains such as Sudan IV or fluorol yellow, which target the aliphatic constituents of the polymer [27,28] and cannot capture early suberization events when suberizing walls accumulate ferulates, presumably playing a role in coupling the aromatic and aliphatic domains.

**Figure 1:**
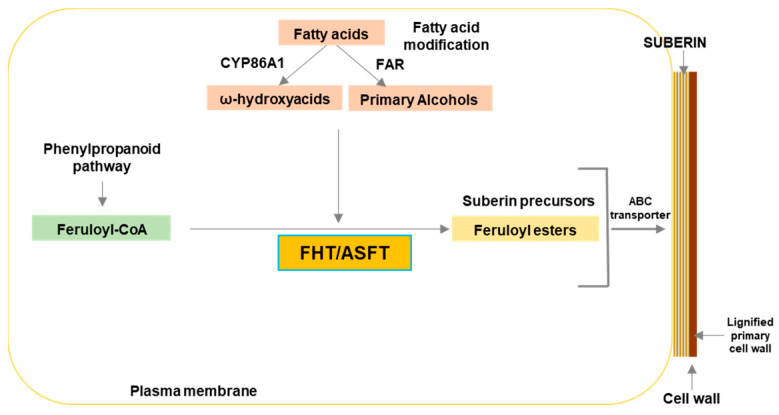
Schematic representation of the enzymatic function of suberin feruloyl transferase (FHT). FHT catalyzes the conjugation of feruloyl-CoA to aliphatic chains such as ω-hydroxy acids and primary alcohols to form feruloyl esters [19,20,21], potentially acting as a linker of suberin to lignin-like or other cell wall polymers.

FHT is a key acyltransferase of the suberin biosynthesis pathway which catalyzes the conjugation of feruloyl-CoA and oxidized fatty acids, both of which are suberin monomers [19,20,21,29]. Hence, it acts as a linker of phenolic to aliphatic compounds to build the suberin polyester (Figure 1). The *FHT* gene encodes an acyltransferase of the BAHD superfamily named after the first four biochemically characterized enzymes of the group, which are plant-specific enzymes that catalyze the transfer of coenzyme A-activated donors onto various acceptor molecules [30].

Here, we present a method that allows precise localization of early suberization sites using the promoter of tomato suberin feruloyl transferase (FHT) fused to GUS (β-glucuronidase). The aim of this study is to determine whether the sites of FHT promoter activation correspond to those of ferulate deposition and thus can function as a robust marker for early suberization events. Transgenic tomato plants expressing *Pro_SlFHT_::GUS* showed ferulate deposition at the position of promoter induction, visualized using alkali UV microscopy and in accordance with the predicted SlFHT functions. This tool can be used as an efficient marker of early suberization events during plant development and stress responses. Such markers will aid in the fundamental understanding of the suberization process and germplasm screening for varietal improvement programs in tomato against diverse stress responses. 

## 2. Results

### 2.1. FHT Proteins of Different Plant Species Have a Conserved HxxxD Motif Involved in Catalysis and a DFGWG Motif Located at the C-Terminal End

**Figure 2:**
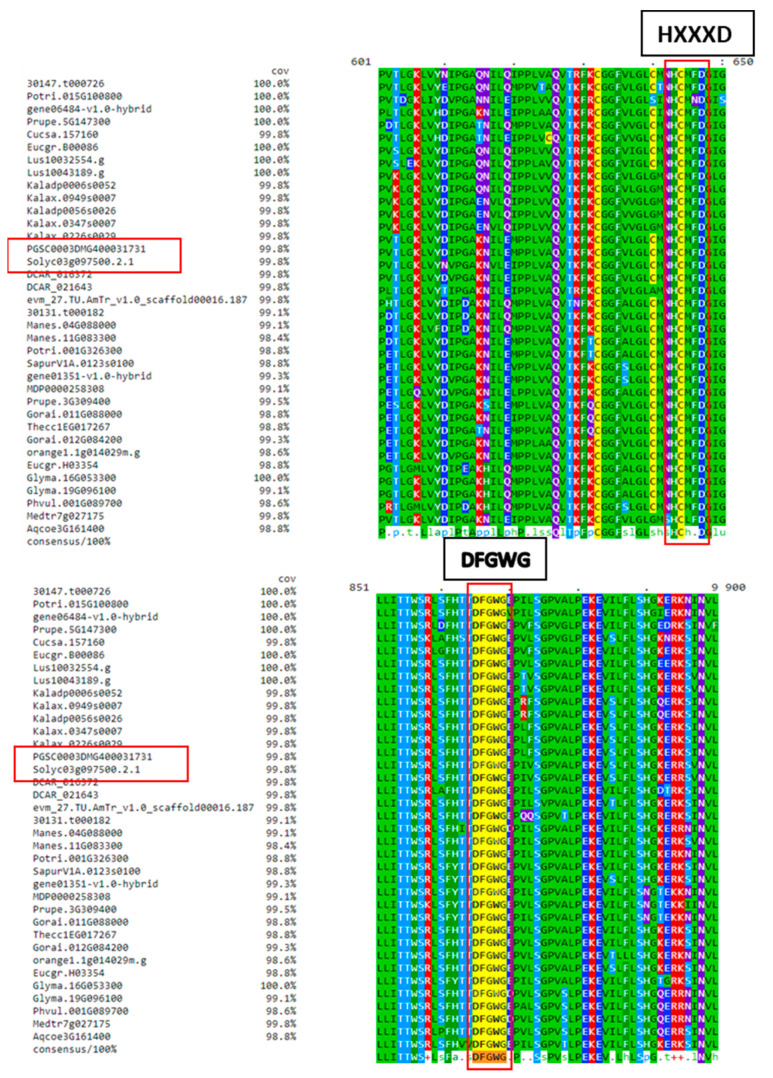
Sequence alignment of feruloyl transferase (FHT) proteins in different plant species. Protein homologs of tomato *FHT* gene (Solyc03g097500) were obtained from www.phytozome.jgi.doe.gov, accessed on 2 May 2023, and matches with more than 85% similarity were used for amino acid sequence alignment in ClustalW and visualized by Mview using BAR (http://bar.utoronto.ca/, accessed on 2 May 2023) webpage. In agreement with the characteristics of BAHD acyltransferases, all FHT proteins of different plant species have conserved HxxxD motif involved in catalysis and DFGWG motif located at the C-terminal end, the latter of which is presumed to have a structural function [21].

In agreement with the characteristics of BAHD acyltransferases, all FHT proteins of different plant species have a conserved HxxxD motif involved in catalysis and a DFGWG motif located at the C-terminal end, the latter of which is presumed to have a structural function [21]. Protein homologs of tomato *FHT* gene (Solyc03g097500) were used for amino acid sequence alignment in ClustalW and visualized by Mview using the BAR (http://bar.utoronto.ca/, accessed on 2 May 2023) webpage (Figure 2). Phylogenetic analysis showed that the tomato *FHT* gene is very close in ancestry to the potato *FHT* (*StFHT*) gene (PGSC0003DMG400031731), which has been characterized in deeper detail [28]. 

### 2.2. Putative Cis Elements Found in Tomato FHT Promoter

The putative *SlFHT* promoter region (1710 bp upstream of the translation initiation) was examined using the PLACE [31] and the PlantCare [32] databases [29]. In agreement with the reported function of the *FHT* gene as a key acyltransferase involved in suberin biosynthesis, sequence analysis showed the presence of cis-regulatory motifs specific to abiotic stresses such as wounding, salt injury, water stress, etc. (e.g., WBOXNTERF3) and biotic stresses such as pathogenesis and salicylic acid receptiveness (e.g., GT1GMSCAM4, WBOXATNPR1). As expected, a number of ABA responsive motifs were present (e.g., WBBOXPCWRKY1, MYB and MYC binding sites, SORLIP1, and WRKY), since suberin biosynthesis is known to be regulated by this hormone [33,34,35]. In addition, motifs corresponding to organ-/cell-/and tissue-specific activation of phenylpropanoid genes (e.g., EBOXBNNAPA) and root and seed inducible motifs were present (e.g., ROOTMOTIFTAPOX1, RYREPEATBNNAPA) (Table 1).

### 2.3. Induction of Pro_SlFHT_:GUS in Tomato Tissues Undergoing Developmental Suberization

We hypothesized that the tomato *FHT* promoter (*Pro_SlFHT_*) could be used as a good marker to study early suberization at the sites of suberin nucleation in different tissues. Hence, we generated *Pro_SlFHT_::GUS* lines in tomato in order to elucidate the participation of tomato FHT promoter in tissues known to deposit suberin. For this, we used the tomato variety Hawaii 7996 (H7996), which has been shown to induce ligno-suberin vascular coating to restrict infection of the vascular pathogen *Ralstonia solanacearum* [28]. 

**Figure 3:**
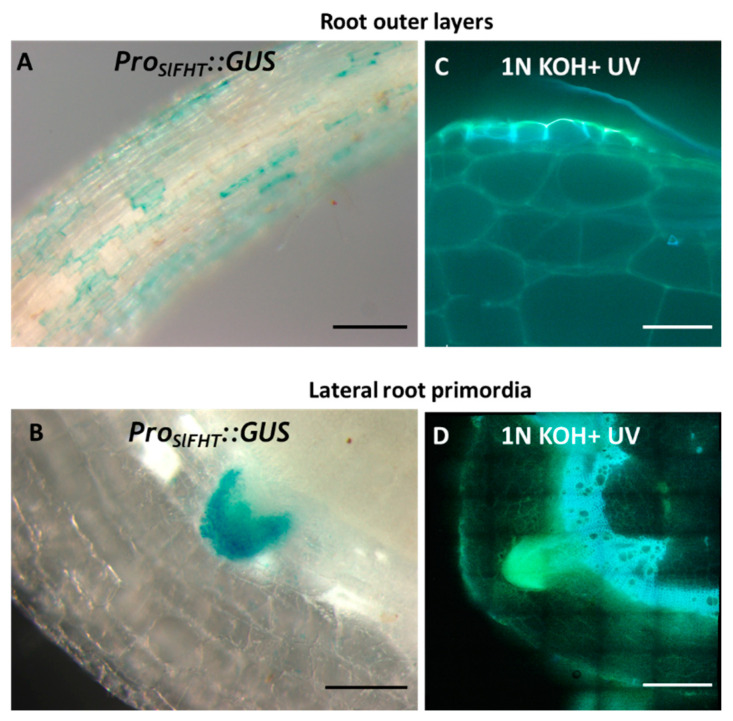
Induction of *Pro_SlFHT_::GUS* in tissues undergoing developmental suberization. Strong induction of *Pro_SlFHT_::GUS* was observed in the (**A**) root outer layers of young seedlings and (**B**) in the emerging lateral root primordia. Correspondingly, in similar samples, the ferulate signal was observed in (**C**) root outer layers of young seedlings and (**D**) lateral root primordia, as observed by alkali UV microscopic technique. Scale bar A = 500 µm, B = 200 µm, C = 40 µm, D = 500 µm.

A fragment consisting of 1713 bp upstream of the initial ATG codon of *SlFHT* was amplified from genomic DNA and fused to the reporter β-glucuronidase (GUS) to generate the *Pro_SlFHT_::GUS* construct, which was transformed into H7996 tomato. In agreement with the critical role reported for FHT/ASFT/RWP in suberization [19,20,21], we clearly observed the induction of *Pro_SlFHT_::GUS* in tissues known to accumulate suberin. Tomato root exodermal cells are known to deposit suberin [36]. We observed strong induction of *Pro_SlFHT_::GUS* in the root outer layers of young seedlings (Figure 3A). Further, prior to emergence, we observed strong induction of *Pro_SlFHT_::GUS* in lateral root primordia, indicating a hardening process by suberin deposition in the cells of the developing lateral root cap (Figure 3B). To analyze whether the induction of *Pro_SlFHT_::GUS* in tomato tissues corresponded to an increase in ferulates, in accordance to the function of FHT, we used a technique whereby ferulates can be detected by emission of blue fluorescence with UV excitation at neutral pH that characteristically changes to a stronger green emission under conditions of high pH such as in the presence of alkali [37,38,39]. We observed that the UV autofluorescence detected in root epidermal cells and lateral root primordia changed from blue to a strong green color upon treatment with alkali (1N KOH pH above 10) (Figure 3C,D), indicating the accumulation of ferulates in these tissues (Figure 3C,D). As expected, this pattern was highly coincidental to the observed induction of *Pro_SlFHT_::GUS*, highlighting the robustness of this method to report dual ferulate/FHT promoter activation.

### 2.4. Induction of Pro_SlFHT_:GUS in Tomato Tissues Undergoing Wound Healing

**Figure 4:**
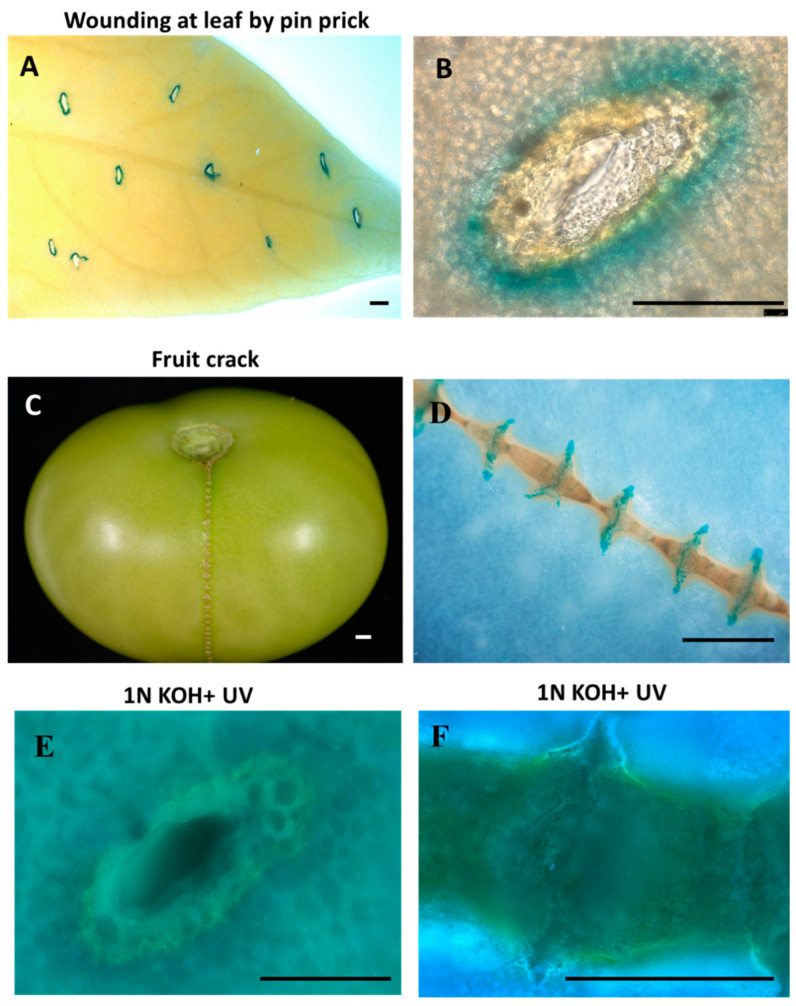
*Pro_SlFHT_::GUS* is induced in leaves and fruits of tomato during wound healing. (**A**) *Pro_SlFHT_::GUS* expression 48 h after pin-prick injury on the leaves. (**C**) Magnified image from (**A**). (**B**) Induction of *Pro_SlFHT_::GUS* was observed in fruit cracks undergoing wound healing. (**D**) Magnified view of the crack showing GUS signal. Correspondingly, ferulate signal was observed in adjacent areas of (**E**) pin-prick injury on the leaves and (**F**) fruit cracks undergoing wound healing, as observed by alkali UV microscopic technique. Scale bar A = 400 µm, B = 200 µm, C = 20 µm, D = 20 µm, E = 200 µm, F = 20 µm.

Since suberin deposition is known to occur as part of the wound-healing response [29,40], we analyzed the induction of *Pro_SlFHT_::GUS* upon injury. At 48 h post pin-prick injury on leaves, a strong induction of *Pro_SlFHT_::GUS* was observed surrounding the injured region (Figure 4A,B). Further, when water imbalance or other factors lead to fruit cracks in tomato, the plants have a mechanism to seal this crack to prevent rotting due to the growth of saprophytes. We observed specific induction of *Pro_SlFHT_::GUS* in the sealing region of the cracks, indicating suberization in this particular wound-healing response (Figure 4C,D). Further, we visualized ferulate deposition using alkali UV microscopy concomitantly with FHT promoter activation (GUS signal) during pin-prick injury and fruit cracks in tomato (Figure 4E,F).

### 2.5. Induction of Pro_SlFHT_::GUS in Vascular Suberization Response in Tomato against Pathogens

**Figure 5:**
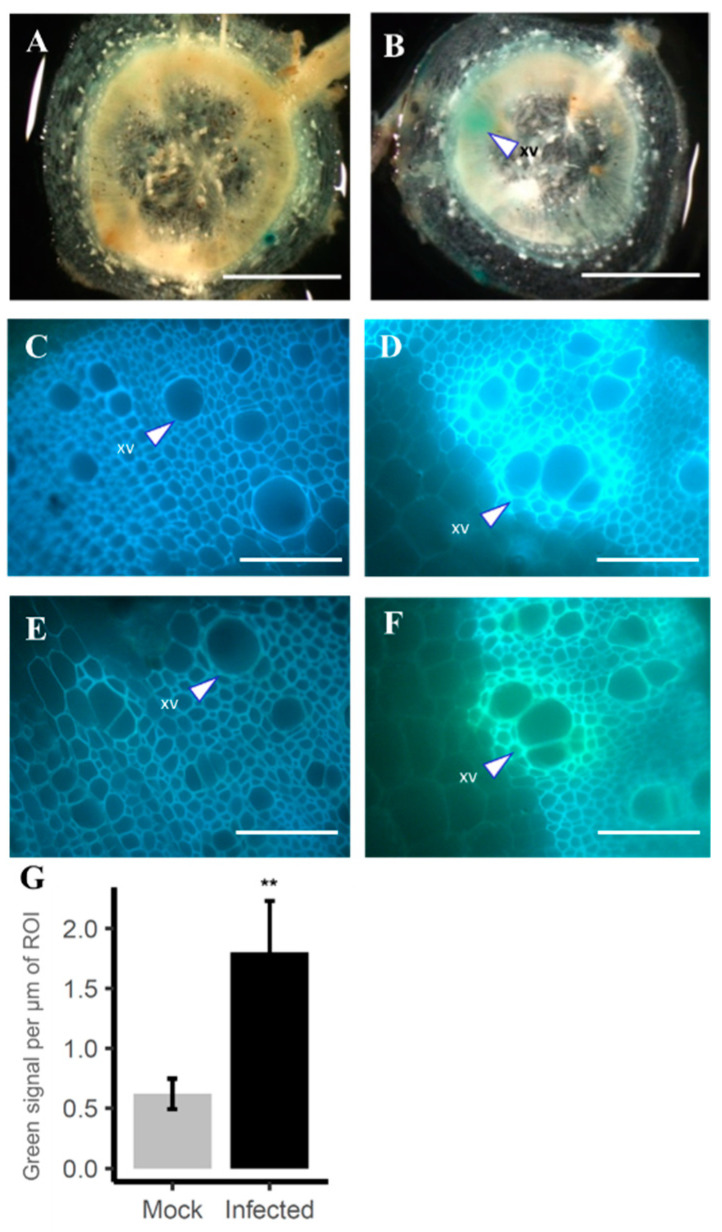
Pathogen-triggered induction of *Pro_SlFHT_::GUS* in xylem vasculature of tomato. *Pro_SlFHT_::GUS* transgenic tomato plants were inoculated through their roots by soaking the soil with 40 mL of suspension per plant of *R. solanacearum* with a concentration of ~1 × 10^7^ CFU mL^−1^ and grown at 28 °C or with water as a control. GUS staining of (**A**) water-treated plants and (**B**) infected plants, where induction of *Pro_SlFHT_::GUS* could be observed in xylem vascular tissue (xv). UV autofluorescence before alkali treatment as observed in the vasculature of water-treated (**C**) or inoculated (**D**). Alkali UV microscopic technique was used to detect ferulate deposition in the vasculature of water-treated (**E**) or inoculated (**F**) samples at 20 days post-inoculation. Scale bar A, B = 2 mm; C, D, E, F = 150 µm. (**G**) Quantification of green fluorescence signal per μm in Region of Interest (ROI) from ferulate deposits upon alkaline treatment with KOH. Green color intensity calculation was performed with ImageJ software by selecting the vascular areas around the main vessels with localized fluorescence or green signal. Images from a representative experiment out of three with *n* = 5 plants per condition. Asterisks indicate statistically significant differences (**, *p*-value < 0.005, α  =  0.05, Fisher’s least significant difference test).

Suberin vascular coating in response to the vascular pathogen *Ralstonia solanacearum* infection has been observed in the resistant tomato cultivar H7996 [28]. *Pro_SlFHT_::GUS* transgenic H7996 tomato plants were inoculated through their roots by soaking the soil with *R. solanacearum* with a concentration of ~1 × 10^7^ CFU mL^−1^ and grown at 28 °C for 20 days. In water-treated plants, induction of *Pro_SLFHT_::GUS* was not observed in the xylem vasculature (Figure 5A). In infected resistant tomato plants, induction of *Pro_SlFHT_::GUS* was observed in the xylem vascular tissue as well as in the outer layers of the root (Figure 5B). Interestingly, we observed that the UV autofluorescence detected in vascular coatings in response to *R. solanacearum* infection in resistant H7996 (Figure 5D) changed from blue to a strong green color upon treatment with alkali (1N KOH pH above 10)—but not in response to mock treatment (Figure 5C)—indicating the presence of ferulates in these coatings (Figure 5F,G), as previously reported [28]. In contrast, no such vascular coating was observed in water-treated plants (non-infected) (Figure 5E,G). These data indicate that resistance to *R. solanacearum* in H7996 is mediated by induction of the *FHT* expression in the vasculature, which is accompanied by ferulate deposition in the same area. The lipophyllic fluorescent dye fluorol yellow, which stains the aliphatic portion of suberin, was also used in *Pro_SLFHT_::GUS* transgenic tomato plants infected with *R. solanacearum* and mock-treated plants. As shown in Figure S1, infection with *R. solanacearum* resulted in a clear increase in root fluorescence compared to mock-treated roots. However, this dye provided less zonal information regarding potential sites of *R. solancearum*-induced suberin deposition in tomato roots.

## 3. Discussion

Suberin deposition as a response to multiple stresses has recently been explored with renewed vigor to develop plants resilient to abiotic and biotic stresses [2]. In tomato, suberin plays vital roles against diverse environmental stresses [41]. However, the absence of markers or tools for localizing suberizing tissues remains as an impediment for the fundamental understanding of the process in tomato and other crop plants, particularly during the response to various stresses, as well as during germplasm screening for varietal improvement programs. In tomato, the visualization of suberin in tissues mostly relies on stains such as Sudan IV or fluorol yellow, which target the aliphatic components [42] and cannot capture the early accumulation of ferulate esters catalyzed by FHT, which is expressed earlier than the fatty acyl biosynthetic machinery [43,44] and before suberin lamellae structuring. Suberin undergoes enormous compositional changes as the lamella matures. In the initial stages, lignin-like polymer is laid down in advance to the suberin polyester [45]. In fact, ferulates constitute a crucial component of the lignin-like polymer, potentially acting as nucleating sites for suberin matrix polymerization [29,46]. Hence, our tool using the promoter of *SlFHT* fused to GUS allows precise localization of early suberization sites. FHT is a key acyltransferase, which catalyzes the conjugation of feruloyl-CoA to aliphatic chains to form feruloyl esters, which are suberin precursors [19,20,21,29]. 

The putative *SlFHT* promoter region showed presence of cis-regulatory motifs specific to abiotic stresses such as wounding, salt injury, water stress, etc. (e.g., WBOXNTERF3) and to biotic stresses such as pathogenesis and salicylic acid receptiveness (e.g., GT1GMSCAM4, WBOXATNPR1), and as expected, a number of ABA responsive motifs were present (e.g., WBBOXPCWRKY1, MYB and MYC binding sites, SORLIP1, and WRKY) since suberin biosynthesis is known to be regulated by ABA [33,34,35]. In addition, motifs corresponding to tissue-specific activation of phenylpropanoid genes (e.g., EBOXBNNAPA) and root and seed inducible motifs were present (e.g., ROOTMOTIFTAPOX1, RYREPEATBNNAPA). The tomato *FHT* gene is very close in ancestry to the potato *FHT* gene [28], and its promoter was also reported to contain similar cis-regulatory motifs [29]. In agreement with the critical role reported for FHT in suberization [17,18,24], we observed the induction of *Pro_SlFHT_::GUS* in the outer root layers, which could correspond to root exodermis, which is well known to deposit suberin [36]. Further, we observed strong induction of *Pro_SlFHT_::GUS* in the lateral root primordia, indicating a hardening process by suberin–ferulate deposition in the cells of the developing lateral root cap before emergence. Interestingly, the root caps of lateral roots have been reported to contain a cuticle-like polymer in Arabidopsis [47]. In contrast, we observed that lateral root primordia initiated suberization during their development in tomatoes. It is possible that both lipid-based polymers, suberin and cutin, having common ancestry [48], perform similar functions towards root cap hardening. However, ferulic acid, which is a chief component of suberin-associated phenolics, is a minor component in the chemically similar cutin polymer [1]. In accordance with the function of FHT, we observed ferulate deposition using alkali UV microscopy at the sites of promoter activation. Since suberin deposition is known to occur as part of the wound-healing response [29,40], we also observed a strong induction of *Pro_SlFHT_::GUS* surrounding the tissues at 48 h post pin-prick injury on leaves. Further, when water imbalance or other factors lead to fruit cracks in tomato, the plants have a mechanism to seal this crack to prevent rotting due to the growth of saprophytes. We could observe specific induction of *Pro_SlFHT_::GUS* in the sealing region of the cracks, indicating suberization in this particular wound-healing response. In all cases, a positive correlation between *FHT* promoter activity and ferulate deposition by alkali UV microscopy was observed. Additionally, induction of *Pro_SlFHT_::GUS* in the vasculature of resistant tomato cultivar H7996 against pathogen *R. solanacearum* was observed. Suberin vascular coating in response to vascular pathogen *R. solanacearum* infection has been previously reported in resistant tomato cultivar H7996 [28]. In infected resistant tomato plants, induction of *Pro_SlFHT_::GUS* was observed in the xylem vascular tissue, and ferulate vascular reinforcements were also observed, showing positive correlation between induction of *Pro_SlFHT_::GUS* and ferulate deposition after infection with *R. solanacearum*. 

Together, these data indicate that this tool can be used as an efficient marker of early suberization events during plant development and stress responses. It can potentially be translated to other vascular pathogens to tomato as well as for studying other stress responses such as salt injury, drought stress, etc. Further, the tomato *FHT* promoter is a good candidate for tissue-specific expression of desired genes and metabolic engineering at targeted sites such as the exodermis, lateral root primordia, wound-healing zones, and other sites undergoing suberization to confer resilience to diverse stresses. In conclusion, we have developed a very effective tool to detect the sites for early suberization events linked to FHT activation and ferulate deposition.

## 4. Materials and Methods

### 4.1. Plant Material and Growth Conditions

The tomato (*Solanum lycopersicum*) variety Hawaii 7996 (H7996) was used for all experiments. Seeds were germinated and plants were grown in pots containing soil (Substrate 2, Klasmann-Deilmann GmbH) mixed with perlite and vermiculite (30:1:1) in controlled growth chambers at 60% humidity and 12 h photoperiod with light intensity of 120–150 µmol·m^−2^·s^−1^. The temperature was set at 27 °C when using LED lighting and at 25 °C when using fluorescent lighting. 

### 4.2. DNA Constructs

The *Pro_FHT_::GUS* construct was generated using the Gateway system (Invitrogen, Waltham, MA, USA). A fragment consisting of 1713 bp upstream of the initial ATG codon (Solyc03g097500) was amplified from genomic DNA of H7996 tomato using the forward primer (ProFHTF1: ACAAGTTTGTACAAAAAAGCAGGCTAAACAACAAAATAAGATTGCAC) and the reverse primer (ProFHTR1: ACCACTTTGTACAAGAAAGCTGGGTTTTCTCAAAATTAATAAATCCTG) containing the attB flanking sequences. This sequence was cloned into the Gateway entry vector pDONR 207 using a BP reaction and then transferred into the Gateway destination vector pGWB3 using an LR reaction. 

### 4.3. Stable Transformation of Tomato

*Pro_FHT_::GUS* construct was transformed into H7996 using *Agrobacterium tumefaciens* strain C58C1. *A. tumefaciens* was used for co-culture with tomato cotyledons. Explant preparation, selection, and regeneration were performed following standard protocols [49]. Transformants were selected on kanamycin-containing MS medium and propagated into subsequent generations. 

### 4.4. Detection of SlFHT Promoter Activity

Plant tissues or taproot–hypocotyl transition zone sections were immersed in an ice-chilled 90% (*v*/*v*) acetone bath and incubated for 20 min on ice, after which they were rinsed with water. Tissues were vacuum-infiltrated for 20 min with a solution containing 1 mM 5-bromo-4-chloro-3-indolyl-beta-D-glucuronic acid (X-Gluc), 50 mM sodium phosphate buffer (pH 7), 1 mM potassium ferrocyanide, 1 mM potassium ferricyanide, and 0.05% (*v*/*v*) Triton X-100. Samples were then incubated at 37 °C for a maximum of 48 h. Stained tissues were washed 2–3 times with phosphate-buffered saline (PBS) and then cleared with 70% (*v*/*v*) ethanol and stored in 70% (*v*/*v*) ethanol. Images were obtained using an Olympus SZX16 stereomicroscope equipped with a DP71 camera system.

### 4.5. Histological Methods

Thin tangential sections were made with a sterile razor blade of leaf discs, fruit section, or taproot–hypocotyl transition zone for histological assays. Autofluorescence from ferulates bound to the cell wall shows a pH-dependent blue-to-green color conversion [37,38,39]. Ferulates in the xylem vascular tissue were visualized by mounting cross-sections in 70% ethanol (neutral pH) and illuminating them with UV with excitation bandpass filter in the range 340–380 nm to observe blue autofluorescence. These same sections were subsequently mounted in 1N KOH (pH above 10) to observe green autofluorescence from ferulates. Green color intensity calculation was performed with ImageJ software by selecting the vascular areas around the main vessels with localized fluorescence or green signal.

### 4.6. Wounding in Leaves

Injuries in leaves were performed by pin prick using a sterile 0.3 × 13 mm needle (30G × ½”, BD Microlance, Becton Dickinson, Franklin Lakes, NJ, USA).

### 4.7. Bacterial Inoculation in Plants

Four- to five-week-old tomato plants were inoculated through their roots with *Ralstonia solanacearum* GMI1000 using the soil drenching method [50]. For this, roots were wounded by making four holes in the soil at the corners of the pot with a 1 mL pipette tip and inoculated with a 1 × 10^7^ CFU mL^−1^ (OD_600_ = 0.01) suspension of bacteria [50]. Inoculated plants were kept in a growth chamber at 28 °C for 20 days.

## Figures and Tables

**Table 1:** Putative cis elements found in tomato FHT promoter.

Group	Motif Name	Sequence	Motif	Function
			*Nº*	
Stress				
	WBOXNTCHN48	CTGACY	1	“W box.” Elicitor-responsive element
	WBOXNTERF3	TGACY	5	“W box.” Response to wounding
	OSE1ROOTNODULE	AAAGAT	5	Organ-specific elements (OSE) in infected cells of root nodules
	OSE2ROOTNODULE	CTCTT	2	Organ-specific elements (OSE) in infected cells of root nodules
	PREATPRODH	ACTCAT	1	Pro- or hypo-osmolarity-responsive element
	RAV1AAT	CAACA	5	RAV1 binding site, cold responsiveness
	MYB1AT	WAACCA	3	Element involved in dehydration responsiveness
	MYBCORE	CNGTTR	2	Element involved in response to water stress
	GT1GMSCAM4	GAAAAA	9	Pathogenesis and salt-induced element
	CURECORECR	GTAC	10	Copper-response element
	CCAATBOX1	CCAAT	5	HSE (heat shock element)
	ANAERO1CONSENSUS	AAACAAA	2	Response to anaerobiosis
	ACGTATERD1	ACGT	4	Involved in etiolation-induced dehydration
Hormones				
ABA	WBBOXPCWRKY1	TTTGACY	1	WRKY proteins binding site, responsive to ABA
	MYB2CONSENSUSAT	YAACKG	1	MYB recognition site involved in dehydration and ABA response
	MYCATERD1	CATGTG	1	MYC binding site involved in response to dehydration and ABA
	MYCATRD22	CACATG	1	MYC binding site involved in response to dehydration and ABA
	MYCCONSENSUSAT	CANNTG	6	MYC binding site involved in response to dehydration and ABA
	SORLIP1AT	GCCAC	4	Light-inducible, root-specific, and ABA-responsive element
Salicylic acid	WBOXATNPR1	TTGAC	3	“W-box”; binding site for SA-induced WRKY transcription factor
Ethylene	ERELEE4 CGTCA-motif	AWTTCAAA	1	Ethylene-responsive element
Auxin	CATATGGMSAUR	CATATG	2	Element involved in auxin responsiveness
Gibberellin	GARE2OSREP1	TAACGTA	1	Gibberellin-responsive element (GARE)
	GAREAT	TAACAAR	2	GARE (GA-responsive element)
	MYBGAHV	TAACAAA	2	Central element of gibberellin (GA) response complex (GARC)
	PYRIMIDINEBOXOSRAMY1A	CCTTTT	2	Gibberellin-response cis element of GARE and pyrimidine
	TATCCACHVAL21	TATCCAC	1	Conserved cis-acting response complex (GARC)
	PYRIMIDINEBOXHVEPB1	TTTTTTCC	2	“Pyrimidine box”. Required for GA induction
Organ-/Tissue-/Cell type-specific				
	ROOTMOTIFTAPOX1	ATATT	12	Root specific element from rolD gene
	SP8BFIBSP8BIB	TACTATT	3	Tuberous root specific element
	RHERPATEXPA7	KCACGW	1	Right part of RHEs (root-hair-specific cis-elements)
	EBOXBNNAPA	CANNTG	6	RRE element. Tissue-specific activation of phenylpropanoids biosynthesis genes
	RYREPEATBNNAPA	CATGCA	1	Required for seed-specific expression
	POLLEN1LELAT52	AGAAA	12	Element required for pollen-specific expression
	CACTFTPPCA1	YACT	28	Element related to mesophyll expression
	BOXIINTPATPB	ATAGAA	3	Conserved element in promoters of plastids genes.
	TAAAGSTKST1	TAAAG	12	Dof1 protein controlling guard cell-specific gene expression
Others				
	MYBPZM	CCWACC	3	Core of consensus myb homolog binding site
	MYBST1	GGATA	2	Core motif of MybSt1 (a potato MYB homolog) binding site
	RYREPEATLEGUMINBOX	CATGCAY	1	RY repeat found in seed-storage protein genes

## Data Availability

The data that support the findings of this study are available from the corresponding author (N.S.C.), upon reasonable request.

## Supplementary Materials

The following supporting information can be downloaded at: https://www.mdpi.com/article/10.3390/plants12091890/s1, Figure S1: Fluorol yellow staining for detecting aliphatic suberin in *ProFHT::GUS* transgenic plants treated with mock or inoculated with 10^5^ CFU/mL of *Ralstonia solanacearum* by pin inoculation. Quantification of mean fluorescence signal along the root, n ≥ 6, error bars: SD. *** = *p*-value < 0.005. One-way ANOVA followed by Tukey HSD. Scalebar: 200 μm.
