# Supplementary material for: The Tomato Feruloyl Transferase FHT Promoter Is an Accurate Identifier of Early Development and Stress-Induced Suberization
Source: Plants (Basel). 2023 May 5;12(9):1890. doi: 10.3390/plants12091890 (PMC10181283; doi:10.3390/plants12091890)
Supplement: Supplementary file 1 [file plants-12-01890-s001.zip › plants-2343346-supplementary.pdf]

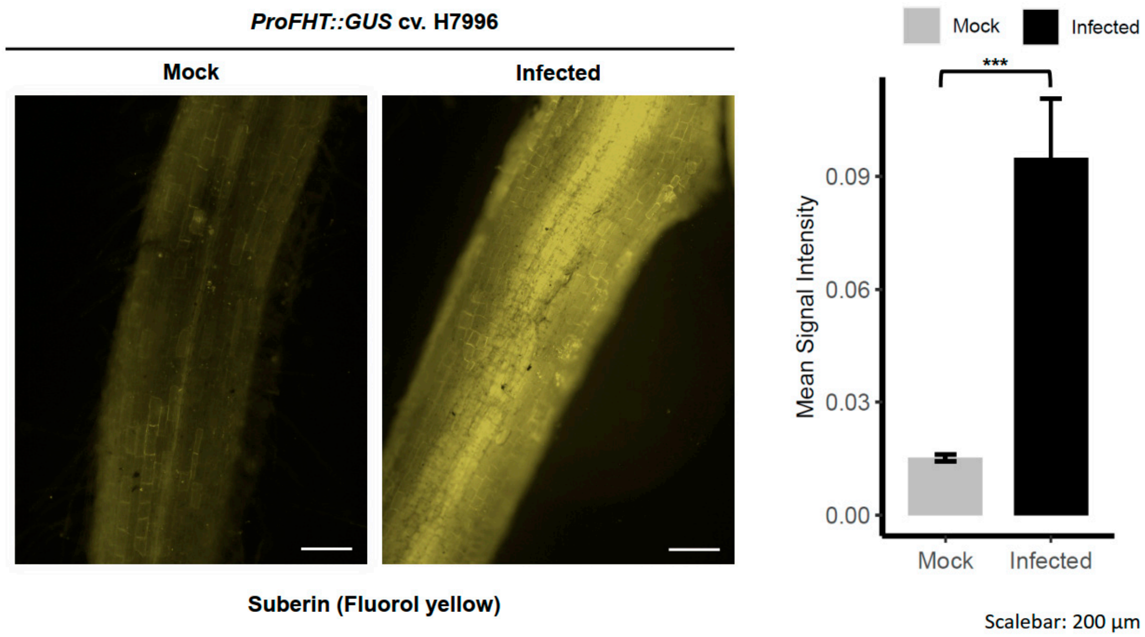

Supplementary Figure S1: Fluorol yellow staining for detecting aliphatic suberin in ProFHT::GUS transgenic plants treated with mock or inoculated with 10^5 CFU/ml of *Ralstonia solanacearum* by pin-inoculation. Quantification of mean fluorescence signal along the root, n ≥ 6, error bars: SD. *** = p-value < 0.005. One-way ANOVA followed by TukeyHSD. Scalebar: 200 µm.
